# Optogenetic control of integrin-matrix interaction

**DOI:** 10.1038/s42003-018-0264-7

**Published:** 2019-01-08

**Authors:** Julia Baaske, Wignand W. D. Mühlhäuser, O. Sascha Yousefi, Sebastian Zanner, Gerald Radziwill, Maximilian Hörner, Wolfgang W. A. Schamel, Wilfried Weber

**Affiliations:** 1 Faculty of Biology, University of Freiburg, Schänzlestr. 1, 79104 Freiburg, Germany; 2 Signalling Research Centers BIOSS and CIBSS, University of Freiburg, Schänzlestr. 18, 79104 Freiburg, Germany; 3 Spemann Graduate School of Biology and Medicine (SGBM), University of Freiburg, Albertstr. 19A, 79104 Freiburg, Germany; 4 Center for Chronic Immunodeficiency, Medical Center—University of Freiburg, Faculty of Medicine, University of Freiburg, 79106 Freiburg, Germany

## Abstract

Optogenetic approaches have gathered momentum in precisely modulating and interrogating cellular signalling and gene expression. The use of optogenetics on the outer cell surface to interrogate how cells receive stimuli from their environment, however, has so far not reached its full potential. Here we demonstrate the development of an optogenetically regulated membrane receptor-ligand pair exemplified by the optically responsive interaction of an integrin receptor with the extracellular matrix. The system is based on an integrin engineered with a phytochrome-interacting factor domain (OptoIntegrin) and a red light-switchable phytochrome B-functionalized matrix (OptoMatrix). This optogenetic receptor-ligand pair enables light-inducible and -reversible cell-matrix interaction, as well as the controlled activation of downstream mechanosensory signalling pathways. Pioneering the application of optogenetic switches in the extracellular environment of cells, this OptoMatrix–OptoIntegrin system may serve as a blueprint for rendering matrix–receptor interactions amendable to precise control with light.

## Abstract (web summary)

Julia Baaske et al. present an optogenetically-regulated membrane receptor-ligand pair that enables light-inducible and light-reversible cell-matrix interaction and controlled activation of downstream mechanosensory signalling pathways. They show that optogenetics can be used to control receptor activation using light.

## Introduction

Optogenetics uses light to control protein function in living cells, and has gathered momentum in the analysis and control of biological processes. The use of light to manipulate signalling processes has the major advantage of being minimally invasive and of enabling precise spatiotemporal control. A vast array of optogenetic switches has been designed to control the activity, localization, interaction or degradation of intracellular proteins to steer biological function^1–4^. However, important extracellular processes such as the interaction of cell surface receptors with ligands or with the extracellular matrix represent a field of intensive study^5–10^. We present here a strategy to optically control the interaction of integrin cell surface receptors with the extracellular matrix.

Integrins are a major group of transmembrane, heterodimeric cell adhesion receptors that link the actin cytoskeleton to the extracellular matrix. Upon ligand binding, integrins undergo a change in conformation that triggers recruitment of signalling and cytoskeletal adaptor proteins such as talin, kindlins and paxillin, which ultimately leads to the formation of focal adhesions and the activation of mechanosensory pathways^11^. This conformational change in canonical integrin signalling relies on the ligand binding which in turn leads to a transmission of conformational changes initially from the metal ion-dependent adhesion site, over the swing out of the hybrid domain, to the extension of the ectodomain and subsequent spatial separation of both tails. This induced conformational change exposes effector binding site and hence activates downstream signalling processes^12^.

Integrins have been found to be involved in the regulation of invasion, proliferation and survival of tumour cells, making them prime targets for anti-tumour therapy^13^. In mammals, the integrin family comprises 24 heterodimers formed by the combination of 18 α and 8 β subunits. One of the most extensively studied members of this receptor family is the αVβ3 integrin, which belongs to the RGD subset of integrins that bind extracellular ligands with Arg-Gly-Asp motifs. Integrin αVβ3 has a critical role in angiogenesis during tumour development, and is associated with tumour growth and metastasis^13,14^. The dynamics of integrin engagement plays a key role in determining the biological outcome of integrin activation, but so far there is a paucity of tools that allow for precise control of integrin activation. In order to provide means for analysing integrin signalling with superior precision, we set out to develop an optogenetically-regulated αVβ3-based integrin–ligand pair. To render the interaction between integrin αVβ3 and the extracellular matrix light-inducible we use the red light-responsive phytochrome B (PhyB) and the phytochrome-interacting factor 6 (PIF6) of *Arabidopsis thaliana*^15^. Light sensing by PhyB is mediated by the photoisomerization of a covalently bound tetrapyrrole chromophore such as phycocyanobilin. Exposure to 660 nm red light leads to an isomerization of the chromophore and the allosteric transition of PhyB from the Pr (inactive) to the Pfr (active) form^16–18^. In its active Pfr form, PhyB binds phytochrome-interacting factors such as PIF6. Illumination with 740 nm far-red light triggers photoconversion back to the inactive Pr form, leading to dissociation from PIF6.

## Results

### Design of a light-inducible membrane receptor–ligand pair

**Fig. 1:**
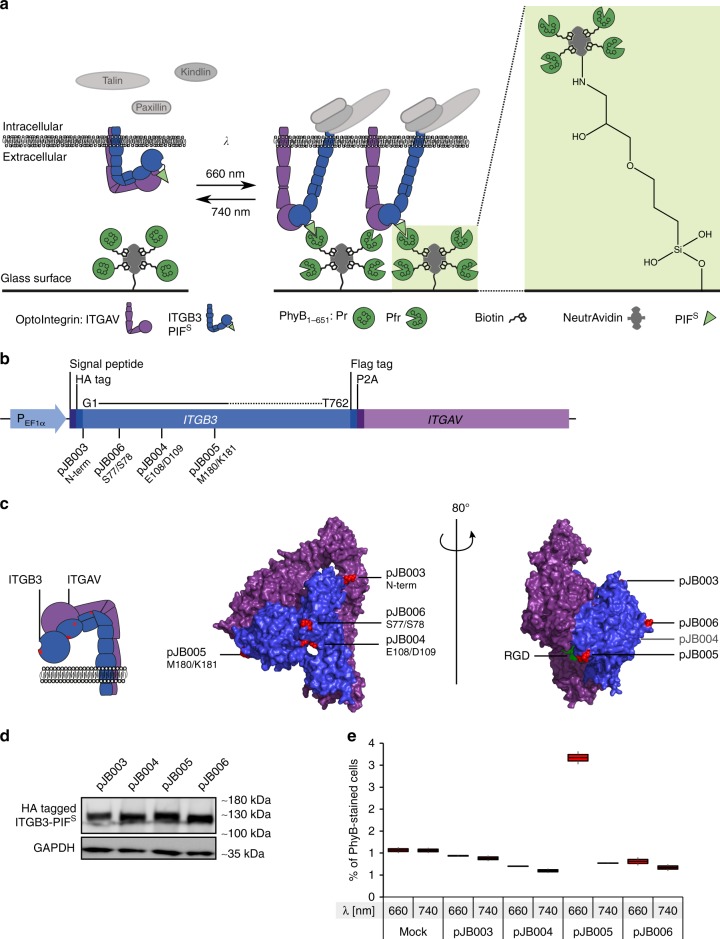
Design and construction of light-inducible receptor–ligand interactions. **a** (3-glycidyloxypropyl)trimethoxysilane (GLYMO)-functionalized glass slides are coated with NeutrAvidin and subsequently with biotinylated PhyB_1–651_. Cells expressing PIF^S^-coupled OptoIntegrins are seeded on top of glass slides. Upon illumination with 660 nm light, PhyB_1–651_ converts from its inactive Pr form to the active Pfr form. The active PhyB_1–651_ form interacts with the OptoIntegrin which then activates mechanosensory pathways. **b** Schematic view of the OptoIntegrin-encoding sequence with PIF^S^ insertion sites. Amino acids numbers under plasmid names refer to insertion sites in the crystal structure (Protein data bank accession number: 1L5G^21^). **c** Crystal structure of αVβ3 extracellular domain with RGD ligand (1L5G^21^). ITGB3 subunit in blue and ITGAV subunit in purple. PIF^S^ insertion sites for different constructs are marked in red. **d** Expression of different OptoIntegrin constructs in HEK-293T cells. Western blot analysis for presence of HA-tagged β subunit in cell lysate expressing different integrin constructs. **e** PhyB_1–651_ binding to surface of untransfected (Mock) HEK-293T cells and HEK-293T cells transiently expressing OptoIntegrin constructs under illumination with 660 nm or 740 nm light. Analysis of stained cells with flow cytometry. Data of two replicates (*n* > 5000 cells) per condition is presented in box and whisker plot format

We postulated that integrin–matrix interactions could be rendered light-switchable by immobilizing PhyB (amino acids 1–651^19^) to a matrix and inserting a PIF6 variant into the extracellular domain of the β subunit of integrin αVβ3 (Fig. 1a). Since PIF is inserted in the extracellular integrin domain which is folded in the endoplasmic reticulum and transported to the cell surface via the secretory pathway, we used a secretion-optimized PIF6 variant (termed PIF^S^)^20^ comprising amino acids 1–100 of PIF6^15,16^ in which a glycosylation motif, as well as two cysteines, have been removed by site-directed mutagenesis (C9S/C10S/S37A). We evaluated four sites for PIF^S^ insertion in the β subunit of the human integrin αVβ3: N-terminally (construct pJB003); between Glu108 and Asp109 (pJB004), which is between the hybrid domain and the β-I domain; between Met180 and Lys181 (pJB005), which is in close proximity to the RGD-binding site; and between Ser77 and Ser78 (pJB006), which is in the hybrid domain (Fig. 1b, c, and Supplementary Table 1; all indicated amino acids refer to the crystal structure: 1L5G (Protein data bank accession number))^12,14,21^. In each construct, PIF^S^ was flanked on both sides by a flexible Ser-Ala-Gly linker. Furthermore, the β subunit was linked via a self-cleaving P2A peptide to an αV subunit to ensure equimolar production of each subunit^22^. To facilitate detection, we added an HA tag to the N-terminus and a FLAG tag to the C-terminus of the β subunit.

We first evaluated expression of the integrin constructs. To this end, human embryonic kidney (HEK-293T) cells were transfected with each construct, lysed and analysed by immunoblotting for the presence of the modified β subunit using anti-HA western blotting (Fig. 1d). The presence of the HA tag confirmed that each β subunit variant is produced in the cells. To further analyse the constructs, it is important to know whether the chimeric PIF^S^ constructs can fold properly and translocate to the plasma membrane, and whether PIF^S^ is accessible for the interaction with PhyB_1–651_. Previous work has shown that only correctly formed integrin heterodimers are transported to the plasma membrane^23^. We stained living HEK-293T cells expressing the integrin variants with anti-HA antibodies and analysed the cells by flow cytometry. All engineered αVβ3 constructs were expressed on the cell surface (Supplementary Figure 1a). To evaluate whether PIF^S^ was accessible for interaction with PhyB_1–651_, we took advantage of the fluorescence of PhyB_1–651_ (Supplementary Figure 2). We incubated transiently transfected HEK-293T cells with PhyB_1–651_ under 660 nm red light or 740 nm far-red light and subsequently measured the PhyB_1–651_ fluorescence intensity using flow cytometry (Fig. 1e and Supplementary Figure 1b). Interestingly, only cells expressing construct pJB005 showed increased fluorescence intensity after 660 nm illumination, indicating that even though all constructs were expressed, only integrin variant from pJB005 had PIF^S^ inserted in a way that it could interact with PhyB_1–651_. Here, we name the αVβ3-PIF^S^ variant of pJB005 OptoIntegrin. Next, we generated HEK-293T, human cervical cancer cell (HeLa) and human breast cancer cell (MCF7) lines which stably express OptoIntegrin αVβ3 in order to further characterize its functionality (Supplementary Figure 3). As control, we further constructed a HEK-293T-based cell line expressing a moxGFP-PIF^S^ fusion on the cell surface in order to differentiate between effects mediated by mere cell–matrix attachment or by functional integrin–matrix interaction (Supplementary Figure 4). We showed that functional PIF^S^ was displayed on the cell surface and interacted with PhyB_1–651_ in a light-dependent manner (Supplementary Figure 4b, c).

### Light controlled cell–matrix interaction

To evaluate whether the interaction of cells expressing OptoIntegrin αVβ3 with a synthetic matrix can be controlled by light, we synthesized PhyB_1–651_-coated glass slides (‘OptoMatrix’). Glass slides were first functionalized with (3-glycidyloxypropyl)trimethoxysilane (GLYMO) and subsequently with NeutrAvidin^24^. Biotinylated PhyB_1–651_ was then added to couple to NeutrAvidin. This treatment prevented wild type cells from attaching and spreading on the glass slide (Supplementary Figure 5a).

**Fig. 2:**
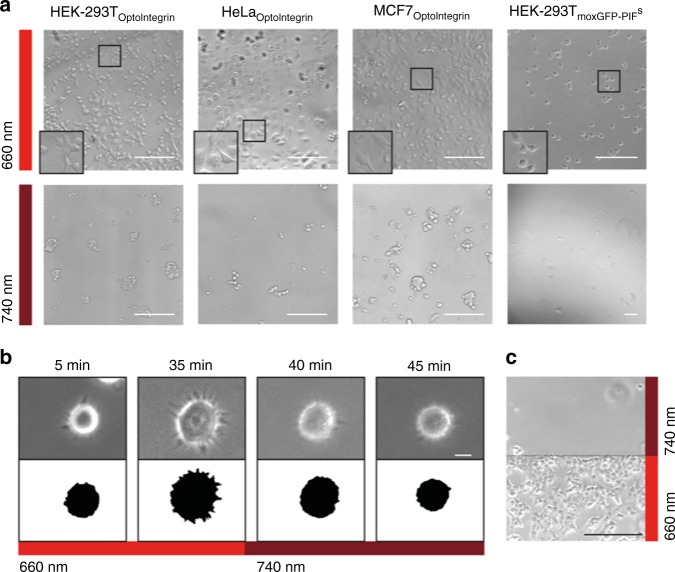
Validation of cell–matrix interaction with different cell lines stably expressing OptoIntegrin. **a** HEK-293T, HeLa and MCF7 cells stably expressing OptoIntegrin or moxGFP-PIF^S^ were seeded on PhyB_1–651_-coated glass slides and incubated under 660 nm or 740 nm light (I = 20 µmol m^−2^ s^−1^) for 5 h and subsequently imaged using a transmission light microscope (scale bar = 200 µm). **b** Live cell imaging of cell–matrix interaction under 660 nm light and then switch to 740 nm light after 35 min with binary images of cell shapes to illustrate the cells spreading. For this, HeLa cells stably expressing OptoIntegrin were seeded on OptoMatrix and subsequently imaged. Micrographs were taken at indicated time points (scale bar = 10 µm). **c** Spatial control of cell attachment with OptoIntegrin-expressing HEK-293T cells. OptoIntegrin-expressing cells were cultivated on OptoMatrix, locally illuminated with 660 nm or 740 nm for 3 min and then left in darkness for 4 h. Afterwards, cells were fixed and imaged (scale bar = 200 µm)

To analyse light-inducible receptor–ligand interactions, we seeded HEK-293T, HeLa and MCF-7 cells stably expressing the OptoIntegrin onto the OptoMatrix under 660 nm or 740 nm light. Under 660 nm light, the cells adhered and formed protrusions, whereas cells kept in 740 nm light did not attach to the slide, but rather formed large aggregates (Fig. 2a). When removing the PhyB surface of OptoMatrix by scratching, however, OptoIntegrin-expressing cells also attached under 740 nm light (Supplementary Figure 5b). The control cells expressing moxGFP-PIF^S^ showed attachment to the OptoMatrix under 660 nm light, yet the cells mainly kept a round shape and did not start to spread. This suggests that mere cell attachment is not sufficient for triggering processes required for efficient spreading. We next compared cell spreading of wildtype cells grown on fibronectin-coated glass slides to OptoIntegrin-expressing cells growing on OptoMatrix (Supplementary Figure 5c). This analysis revealed no significant difference in cell area (p-values obtained using Welch-test: *p* = 0.184 for HEK-293T, *p* = 0.878 for HeLa and *p* = 0.124 for MCF7, see Supplementary Figure 5c).

A strong advantage of using the PhyB-PIF system is the ability to instantaneously reverse an interaction using 740 nm light. In live cell imaging experiments, stably OptoIntegrin-expressing HeLa cells exposed to 660 nm light attached, formed filopodia and spread on the matrix. Upon switching to 740 nm far-red light, however, cells immediately retracted and almost completely detached from OptoMatrix (Fig. 2b, Supplementary Movies 1 (HeLa), 2 (MCF7), 3 (HEK-293T)). Besides temporal control, light also enables high spatial control. To analyse spatially controlled cell attachment, we illuminated one half of the OptoMatrix surface with 660 nm and the other one with 740 nm light (Supplementary Figure 6). Cultivating cells in this configuration revealed attachment and spreading exclusively in the 660 nm-illuminated area (Fig. 2c).

### Adhesion via OptoIntegrin triggers mechanosensing pathways

**Fig. 3:**
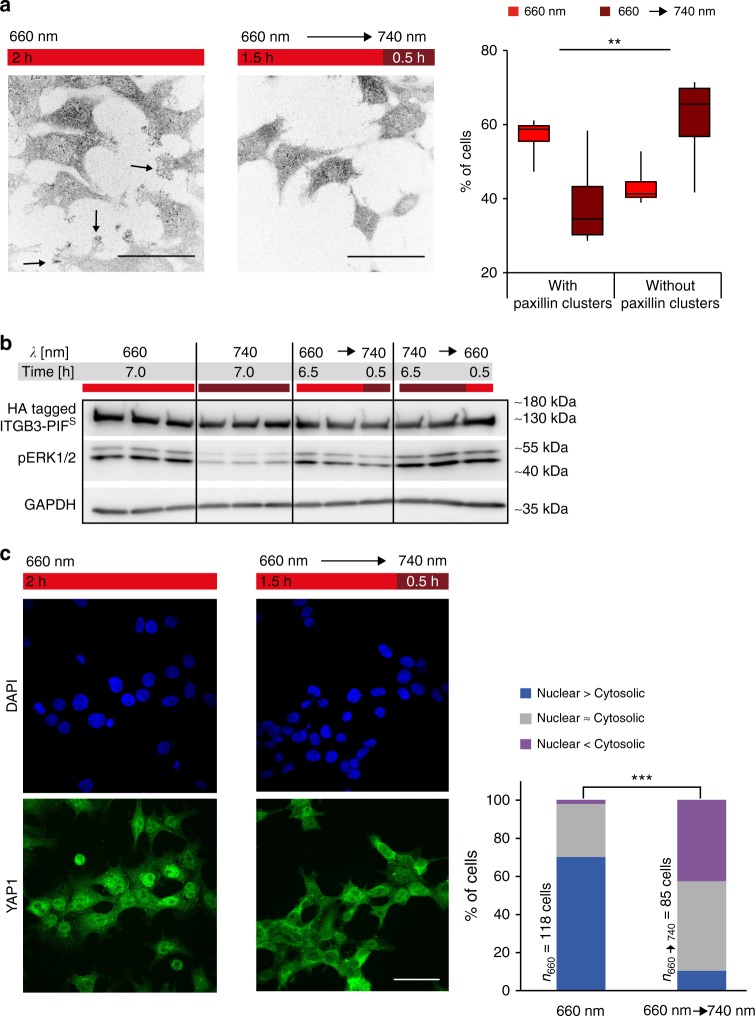
Transducing signals from the extracellular to the intracellular with cells expressing OptoIntegrin. Cells were seeded on PhyB_1–651_-coated glass surface and illuminated as indicated. **a** Paxillin staining with anti-paxillin antibody of fixed stably OptoIntegrin-expressing HEK-293T cells. The intensities of the images were inverted to better visualize clusters at cell edges. The data of three independent experiments where at least 35 cells per condition were analysed in a blinded manner is presented in box and whisker plot format (*p* = 0.000989 using Cochran–Mantel–Haenszel-test with one degree of freedom, showing a consistent difference in the populations across several repeats). **b** Phosphorylation of ERK1/2 in stably OptoIntegrin-expressing HEK-293T cells in response to different light conditions analysed by western blot. Biological triplicates are shown for each condition using anti-pERK1/2 antibodies and anti-HA antibodies to detect pERK1/2 and OptoIntegrin HA tagged β subunit, respectively. **c** YAP1 (green) and nucleus (DAPI, blue) staining of OptoIntegrin-expressing HEK-293T cells. Chart shows the percentage of cells from a single experiment where YAP1 is localized mainly in the cytoplasm, mainly in the nucleus or in an equal distributions between cytoplasm and nucleus (*p* < 0.0001, with a Chi-square value of 87.56 and two degrees of freedom, using Chi-square test of independence shows dependent variables). For (**a**), (**b**) and (**c**) λ_1/2_ = 660/740 nm, I_1/2_ = 20 µmol m^−2^ s^−1^ and for **a** and **b** scale bar = 50 µm

Ligand binding of integrins leads to subsequent clustering and the formation of focal adhesions, which not only mechanically links the matrix-bound integrins to the cytoskeleton, but also engages mechanosensory signalling pathways^11^. These macromolecular assemblies are stabilized through the recruitment of several proteins such as paxillin, which is an important scaffolding protein containing several protein interaction domains^25^. We next assessed whether the OptoIntegrin-OptoMatrix-mediated interaction could trigger such intracellular mechanosensory pathways. To this aim, HEK-293T cells stably expressing OptoIntegrin were allowed to attach to PhyB_1–651_-coated glass slides for 1.5 h under 660 nm light. Two light treatments were then applied: one group of cells was switched from 660 nm to 740 nm for 0.5 h, while the other group was kept under constant 660 nm light. Cells were subsequently fixed, stained for paxillin and imaged (Fig. 3a and Supplementary Figure 7a). In cells grown under constant 660 nm light, we observed in approximately 55% of the cells paxillin clusters at the edges. After switching to 740 nm light, cells were less spread and only 40% of cells showed paxillin cluster formation, which was significantly different compared to constant 660 nm light condition. Similar results were further obtained using HeLa cells stably expressing OptoIntegrin (Supplementary Figure 7b–d). In approximately 80% of the HeLa cells illuminated with constant 660 nm light, we observed clustering of paxillin at the cellular periphery. In contrast, only approximately 20% of cells treated with far-red light showed this distinct paxillin localization pattern. These observations suggest that engineered OptoIntegrin−OptoMatrix interactions, and the ensuing cellular formation of focal adhesions, can be induced and reversed by light.

After demonstrating the recruitment of direct integrin interaction proteins, we next examined whether mechanosensory signalling events further downstream could be controlled using this light-inducible OptoIntegrin–OptoMatrix interaction. First, we examined the activation of the Ras-Raf-MEK-ERK cascade, which is activated downstream of integrins^26^. For this we set up two constant light conditions in which we incubated cells stably expressing OptoIntegrin under constant 660 nm or 740 nm light and two conditions where the light colour was switched 30 min before cell lysis (Fig. 3b). Cells were analysed by immunoblotting for the presence of active, phosphorylated form of ERK1/2 (pERK1/2). Cells stably expressing OptoIntegrins showed higher abundance of pERK1/2 under 660 nm illumination compared to 740 nm, indicating that OptoIntegrins activate intracellular signalling upon ligand binding. Disruption of the OptoIntegrin–PhyB_1-651_ interaction induced by switching from 660 nm to 740 nm led to a reduction of pERK1/2 levels. Cells expressing moxGFP-PIF^S^ grown on the OptoMatrix, as well as wildtype cells and OptoIntegrin-expressing cells grown on fibronectin-coated glass slides did not show such difference under the different illumination conditions (Supplementary Figure 8a).

Further, we speculated that by triggering OptoIntegrin activation, we could mimic the mechanical stimuli that activate the transcriptional regulator YAP1 (Yes-associated protein 1). Activation of YAP1 is regulated by cytoskeletal tension and substrate stiffness and results in the nuclear translocation of the protein^27–30^. To this end, stably OptoIntegrin-expressing HEK-293T cells were allowed to interact with the OptoMatrix for 1.5 h under 660 nm red light, and were subsequently exposed to 740 nm far-red light or kept under 660 nm light for additional 30 min. Cells kept under 660 nm light showed higher abundance of nuclear YAP1, whereas cells that were switched to 740 nm light displayed a more diffuse localization of YAP1 (Fig. 3c and Supplementary Figure 8b). Cells grown on fibronectin-coated glass slides did not show light-dependent differential YAP1 localisation (Supplementary Figure 8c). Analysis of YAP1 localization in HeLa cells expressing the OptoIntegrin showed results similar to HEK-293T cells (Supplementary Figure 8d). These results suggest that OptoIntegrin is able to induce cell–matrix interactions and transduce mechanical stimuli.

### Conclusions

The activation of our OptoIntegrin is likely regulated through a pulling force created by the interaction of PhyB_1–651_ and PIF^S^. We hypothesize that this interaction mimics the natural ligand binding by creating an adhesion force that pulls the integrin into the extended, active conformation. Through this force generation, we suggest, that we shortcut the canonical adhesion mechanism to provoke an adhesion signal. This light-controlled OptoIntegrin can thus, in the future, be used to study more in detail the integrin activation mechanisms and shed further light on the role of mechanical forces.

Optogenetic methods enable precise control of cellular processes, and are powerful tools to study biological signalling. Here, we expanded the application of optogenetics to the extracellular environment by generating the red light-switchable PhyB OptoMatrix for activation of a PIF^S^-containing OptoIntegrin. Using this system, we showed for the first time, to our knowledge, that it is possible to use optogenetics to faithfully recapitulate an extracellular ligand–receptor interaction that transduces signals from the matrix to the intracellular mechanosensory machinery of cells. The extension of this system to other ligand–receptor interactions, as well as the design of specialised OptoMatrices and OptoLigands, could provide a straightforward, widely applicable method to exert precise spatiotemporal control over receptor activation using light. This technology therefore has the potential to provide unprecedented insight into the dynamics and signalling sequelae of receptor activation.

## Methods

### Plasmids

The plasmids designed in this study are described in Supplementary Table 1.

### Sample illumination

Illumination was performed with light boxes similar to those used before (for further information see supplementary methods)^31^, with LED panels emitting at 660 nm (LED660N-03, Roithner LaserTechnik; LH W5Am, Osram Opto Semiconductors; L-53SR, Kingbright Electronic) or 740 nm (H2A1-H740, Roithner LaserTechnik; LED740-01AU, Roithner LaserTechnik; LZ4-00R308, LED Engin). LEDs for live imaging experiments were pE-4000 from CoolLED. LED-stand was 3D printed in house and equipped with 740 nm (LED740-01AU) and 660 nm LEDs (LED660N-03) with intensities of 2000 µmol m^−2^ s^−1^ and 300 µmol m^−2^ s^−1^, respectively. Light intensities and spectra were measured using an AvaSpec-ULS2048 fiber optic spectrometer (Avantes BV). All cell handling involving the PhyB-based systems was done under safe 520 nm green light.

### PhyB_1–651_ production

Biotinylated PhyB_1–651_ was produced in *Escherichia coli* BL21 (DE3) (Invitrogen) together with the enzymes Ho and PcyA for biosynthesis of phycocyanobilin^32^. PhyB_1–651_ was purified by IMAC. Protein purity and chromophore incorporation were verified by Coomassie staining and 1 mM zinc acetate staining (Supplementary Figure 2a). Protein concentration was determined by Bradford assay (Bio-Rad, cat. no.: 500-0006) using bovine serum albumin (BSA, Sigma Aldrich, cat. no.: 05479) as standard. For flow cytometry staining of cells, PhyB_1–651_ was dialysed against PBS (2.7 mM KCl, 1.5 mM KH_2_PO_4_, 8.1 mM Na_2_HPO_4_, 137 mM NaCl) using SnakeSkin Dialysis Tubes (Fisher Scientific, cat. no.: 10005743).

### Coating glass with PhyB_1–651_

Glass slides (Carl Roth, cat. no.: P231.1) were washed in methanol (MeOH), dried and immersed in a 1:3 (v:v) solution of 30% (w/w) hydrogen peroxide and 95% (w/w) sulfuric acid for 40 min at 70 °C. After washing three times in deionized water and two times in MeOH, the glass slides were immediately immersed in a 5% (v/v) (3-glycidyloxypropyl)trimethoxysilane (GLYMO, Sigma, cat. no.: 440167) solution in MeOH for 16 h at room temperature. The glass slides were subsequently washed three times in 2-propanol and dried at 105 °C for 1 h (after this, glass could be stored in a dry place)^24^. The slides were then coated with 5 mg mL^−1^ NeutrAvidin (Thermo Scientific, cat. no.: 31050, dissolved in PBS) overnight at room temperature (RT), washed three times with PBS, and before final incubation in 3 mg ml^−1^ PhyB_1–651_ (in elution buffer (50 mM NaH_2_PO_4_, 300 mM NaCl, 250 mM imidazole, pH 8.0)) for 2 h at RT in darkness.

### Coating glass with fibronectin

Glass slides (Carl Roth, cat. no.: P231.1) were incubated with 200 µl of 25 mg mL^−1^ fibronectin in PBS for 2 h at RT. After incubation slides were directly transferred to cell culture medium for further use.

### Cell culture and transient transfections

Human embryonic kidney cells (HEK-293T), human cervical cancer cells (HeLa), human breast cancer cells (MCF7) and cell lines stably expressing OptoIntegrin were maintained in Dulbecco’s modified Eagle’s medium (DMEM) complete (PAN, cat. no.: P04-03550) supplemented with 10% (v/v) fetal calf serum (FCS, PAN, cat. no.: P30-3306, lot no.: P140204), 100 U mL^−1^ penicillin and 100 μg mL^−1^ streptomycin (PAN) at 37 °C in a humidified atmosphere containing 5% CO_2_. For passaging, cells were detached enzymatically using a trypsin/ethylenediaminetetraacetic acid (EDTA) solution (PAN, cat. no.: P10-023500), for attachment experiments, cells were detached using non-enzymatic cell dissociation buffer (Sigma Aldrich, cat. no.: C5914) and placed in DMEM supplemented with 1% FCS, 100 U mL^−1^ penicillin and 100 μg mL^−1^ streptomycin (PAN). For transient transfections, HEK-293T and HeLa cells were seeded in DMEM complete to obtain 70–80% confluency at time of transfection. After 24 h, cells were transfected using polyethylenimine (PEI, linear, MW: 25 kDa, Polyscience, Warrington, PA, cat. no.: 23966‐2), as described elsewhere^33^. After 6 h the medium was exchanged with fresh DMEM complete. Following incubation for 24–48 h in the dark, the cells were used for experiments.

### Generation of stable OptoIntegrin cell lines

For production of VSV-G-pseudotyped lentiviral particles, 9 × 10^4^ HEK-293T cells cm^−2^ were seeded in DMEM complete. After 24 h, the cells were transfected using PEI with the packaging plasmids pLTR-G^34^, pCD/NL-BH* ΔΔΔ^35^ and the OptoIntegrin-encoding plasmid pJB021 in a mass ratio of 1:1:2. 5 h after transfection, the medium was replaced by DMEM lenti (Advanced DMEM (Thermo Fisher, cat. no.: 12491015), 2% FCS, 100 U mL^−1^ penicillin and 100 μg mL^−^1 streptomycin, 10 μM cholesterol, 10 μM egg lecithin (Serva Electrophoresis, cat. no.: 27608), 1 × chemical defined lipid concentrate (Thermo Fisher, cat. no.: 11905031))^36^. The supernatant containing the viral particles was harvested after 48 h and filtered through a 0.45 μm C-AS filter (GE Healthcare, cat. no.: 514-1113). Clarified supernatant was added to each well of a 6-well plate containing HEK-293T, HeLa or MCF7 cells at 70–80% confluency. After 72 h, the transduced cells were harvested and stained with primary anti-HA antibody (Sigma, cat. no.: H9658, diluted 1:100 in PBS supplemented with 2% FCS) and subsequently with Alexa Fluor 488-labelled donkey anti-mouse IgG (Thermo Fisher, cat. no.: A2102, diluted 1:200 in PBS containing 2% FCS) for fluorescence-activated cell sorting (S3e Cell Sorter, Bio-Rad). For this, the cells were washed in PBS supplemented with 2% FCS and incubated with anti-HA for 25 min on ice. Subsequently, cells were washed twice with PBS containing 2% FCS and incubated with secondary Alexa Fluor 488-labelled donkey anti-mouse IgG for 25 min on ice. The cells were then washed three times and resuspended in PBS containing 2% FCS for sorting of high expressing population by fluorescence-activated cell sorting (Supplementary Figure 3). For the control cell line expressing pJB031 the same procedure was applied, but cells were not sorted after transduction.

### Flow cytometry staining using PhyB_1–651_

Cells were detached using non-enzymatic cell dissociation buffer. Cells were subsequently washed with PBS supplemented with 10% FCS before incubation in PhyB_1–651_ (1.5 mg mL^−1^) in PBS supplemented with 2% FCS for 1 h at 4 °C. This incubation step was performed under 660 nm or 740 nm light (20 µmol m^−2^ s^−1^). Then cells were washed twice with ice-cold PBS and resuspended in PBS supplemented with 2% FCS. PhyB_1–651_ fluorescence was measured with a GALLIOS flow cytometer (Beckman Coulter) using 638 nm red excitation laser and a 660/20 emission filter.

### Attachment assays

For attachment assays, cells were detached using non-enzymatic cell dissociation solution and seeded on PhyB_1–651_- or fibronectin-coated glass slides and illuminated as indicated. For live cell imaging and spatial control experiments, cells were seeded on PhyB_1–651_ coated glass slides placed in an IBIDI glass bottom dish (cat. no.: 81158), illuminated with 740 nm until imaging started, where cells were subsequently illuminated as indicated and kept at 37 °C and 5% CO_2_. Illumination intensities for live cell imaging ranged for 660 nm between 30 and 60 µmol m^−2^ s^−1^ and for 740 nm from 90–120 µmol m^−2^ s^−1^. Images were taken using either an Axio Observer Z1/7 microscope (Zeiss) with a 20 × objective or an EVOS Xl transmitted light microscope (Fisher Scientific). Binary images of cell shapes were obtained with Fiji^37^. Cell spreading area was determined by dividing pictures into smaller tiles (HEK-293T: 100 × 100 PxI; HeLa/MCF7: 300 × 300 PxI) from which 30 random tiles for HEK-293T and 10 for HeLa/MCF7 were manually segmented to determine cell spreading area. Area was determined using Green formula from OpenCV^38^.

### Immunostaining

For paxillin and YAP1 staining, untransfected or OptoIntegrin-expressing HEK-293T cells were seeded at a density of ~1 × 10^5^ cells cm^−2^ on glass coverslips coated with PhyB_1–651_ or fibronectin, and subsequently illuminated as indicated. HeLa cells were transfected (as described above) with pCMV-LifeAct-TagGFP2 (Ibidi, cat. no.: 60101) 24 h before the experiment and subsequently handled in the same manner. Cells were fixed with 4% (w/v) paraformaldehyde in PBS for 15 min under green light conditions. Following a washing step with PBS, samples were permeabilized with PBS containing 0.5% (v/v) Triton X-100 for 10 min and blocked for 1 h with PBS containing 1% (w/v) BSA before being incubated for 1 h with the anti-paxillin primary antibody (Atlas Antibodies, cat. no.: HPA051309, diluted 1:100 in blocking buffer) or anti-YAP1 primary antibody (Santa Cruz, cat. no.: sc-101199, diluted 1:100 in blocking buffer). After washing with PBS-T (PBS with 0.05% (v/v) Tween-20), samples were incubated for 1 h with an Alexa Fluor 647-labelled goat anti-rabbit IgG (Thermo Scientific, cat. no.: A-21244, diluted 1:200 in blocking buffer) or Alexa Fluor 488-labelled donkey anti-mouse IgG (Thermo Fisher, cat. no.: A2102, diluted 1:200 in blocking buffer) and washed again with PBS-T. Finally, the stained coverslips were mounted on microscope slides with Mowiol 4-88 (Carl Roth, cat. no.: 0713), and confocal images were acquired on an upright confocal microscope (Nikon Instruments Eclipse Ni-E with a C2 confocal laser scanner, 100 × oil objective NA = 1.45 or 60× oil objective NA = 1.40). TagGFP2 and Alexa Fluor 488 or Alexa Fluor 647 were excited with a 488 or 647 nm laser, respectively. Image analysis was performed with Fiji software^37^. Subcellular localization of YAP1 or paxillin was manually evaluated.

### Western blot

Cells were seeded on PhyB_1–651_ coated glass slides at a density of 1.3 × 10^5^ cells cm^−2^ and treated with indicated light conditions. Cells were then immediately lysed using ice-cold lysis buffer (20 mM Tris-HCl, 100 mM NaCl, 1 mM EDTA, 0.5% (v/v) Triton X-100, 0.1% (w/v) SDS) supplemented with protease inhibitors (complete protease inhibitor cocktail, Roche, cat. no.: 04693116001) and phosphatase inhibitors (1 mM sodium orthovanadate, 10 mM sodium pyrophosphate, 50 mM sodium fluoride, 10 mM β-glycerophosphate). For expression study of integrin β subunit cells were lysed 24 h after transfection without any further treatments. After 10 min incubation on ice, lysates were transferred to −80 °C for further lysis. Lysates were thawed on ice and centrifuged at 10,000 × *g* at 4 °C for 10 min. The supernatants were mixed with 5× SDS loading buffer (50% (v/v) glycerol, 312.5 mM Tris, 0.05% (w/v) bromphenol blue, 10% (w/v) SDS, 12.5% (v/v) 2-mercaptoethanol), boiled at 95 °C for 5 min, separated by sodium dodecyl sulfate polyacrylamide gel electrophoresis (SDS-PAGE) and transferred to polyvinylidene fluoride (PVDF) membranes. The membranes were blocked in blocking buffer (TBS-T (TBS (20 mM Tris, 150 mM NaCl, pH 7.4) containing 0.05% (v/v) Tween-20) supplemented with 5% (w/v) BSA (Sigma-Aldrich, cat. no.: 05479)) at RT for 1 h. Primary antibody incubation was conducted at 4 °C overnight or for 1 h at RT in blocking buffer. After three washing steps with TBS-T, the blots were incubated with the corresponding horseradish peroxidase-coupled secondary antibody in blocking buffer at RT for 1 h. Following three washing steps with TBS-T, chemiluminescence was detected with ECL substrate using the ImageQuant LAS-4000 mini system (GE Healthcare, cat. no.: 28-9558-13). For Western blotting the following primary antibodies (diluted 1:1000 in blocking buffer) were used for Western blotting: mouse anti-HA (Sigma, cat. no.: H9658), rabbit anti-pERK1/2 (CST, cat. no.: 9101S), rabbit anti-GAPDH (CST, cat. no.: 5174S), rabbit anti-pFAK Y397 (CST, cat. no.:3283). The following secondary antibodies were used for detection: goat anti-rabbit-HRP (CST, cat. no.: 70745; 1:3000) and goat anti-mouse-HRP (Santa Cruz, cat. no.: sc-2005; 1:5000). All uncropped blots are presented in Supplementary Figures 9 and 10.

### Statistical analysis

Cochran–Mantel–Haenszel-Test (continuity correction: 0.5, χ^2^ with one degree of freedom) was performed to analyse multiple 2 × 2 tables for paxillin staining. The Chi-Square test of independence with two degrees of freedom was used to analyse independent 2 × 3 tables from YAP1 staining. Welch-test was used to determine two test null hypothesis for difference of cell surface areas. Results were considered as significant when *p* < 0.01. For statistical analysis Excel 2013, Python 3.6.6 and GraphPad Prism7 were used.

## Supplementary information

Description of Supplementary Video

Supplementary Movie 1

Supplementary Movie 2

Supplementary Movie 3

Supplementary Information

## Data Availability

The datasets generated during and/or analysed during the current study are available in the Figshare repository: https://figshare.com/s/fc63580c3f69355423cc.

## References

[1] Kolar K, Weber W (2017). Synthetic biological approaches to optogenetically control cell signaling. Curr. Opin. Biotechnol..

[2] Leopold, A. V., Chernov, K. G. & Verkhusha, V. V. Optogenetically controlled protein kinases for regulation of cellular signaling. *Chem. Soc. Rev*. (2018). 10.1039/C7CS00404D. 10.1039/c7cs00404d PMC5882534 29498733

[3] Nihongaki Y, Otabe T, Sato M (2018). Emerging approaches for spatiotemporal control of targeted genome with inducible CRISPR-Cas9. Anal. Chem..

[4] Kolar K, Knobloch C, Stork H, Žnidarič M, Weber W (2018). OptoBase: A web platform for molecular optogenetics. ACS Synth. Biol..

[5] Kadem LF (2017). High-frequency mechanostimulation of cell adhesion. Angew. Chem. - Int. Ed..

[6] Liu Z (2016). Nanoscale optomechanical actuators for controlling mechanotransduction in living cells. Nat. Methods.

[7] Rosales AM, Anseth KS (2016). The design of reversible hydrogels to capture extracellular matrix dynamics. Nat. Rev. Mater..

[8] Valon L, Marín-Llauradó A, Wyatt T, Charras G, Trepat X (2017). Optogenetic control of cellular forces and mechanotransduction. Nat. Commun..

[9] Yüz, S. G., Ricken, J. & Wegner, S. V. Independent control over multiple cell types in space and time using orthogonal blue and red light switchable cell interactions. *Adv. Sci*. 1800446 (2018). 10.1002/advs.201800446. 10.1002/advs.201800446 PMC6097145 30128251

[10] Schmidt D, Tillberg PW, Chen F, Boyden ES (2014). A fully genetically encoded protein architecture for optical control of peptide ligand concentration. Nat. Commun..

[11] Sun Z, Guo SS, Fässler R (2016). Integrin-mediated mechanotransduction. J. Cell Biol..

[12] Campbell ID, Humphries MJ (2017). Integrin structure, activation, and interactions. *Cold Spring Harb*. Prespectives Biol..

[13] Desgrosellier JS, Cheresh DA (2010). Integrins in cancer: biological implications and therapeutic opportunities. Nat. Rev. Cancer.

[14] Dong X (2012). αvβ3 integrin crystal structures and their functional implications. Biochemistry.

[15] Khanna WR (2004). A novel molecular recognition motif necessary for targeting photoactivated phytochrome signaling to specific basic helix-loop-helix transcription factors. Plant Cell.

[16] Levskaya A, Weiner OD, Lim WA, Voigt CA (2009). Spatiotemporal control of cell signalling using a light-switchable protein interaction. Nature.

[17] Quail PH (2002). Phytochrome photosensory signalling networks. Nat. Rev. Mol. Cell Biol..

[18] Burgie ES, Bussell AN, Walker JM, Dubiel K, Vierstra RD (2014). Crystal structure of the photosensing module from a red/far-red light-absorbing plant phytochrome. Proc. Natl. Acad. Sci..

[19] Matsushita T, Mochizuki N, Nagatani A (2003). Dimers of the N-terminal domain of phytochrome B are functional in the nucleus. Nature.

[20] Yousefi, O. S. *et al*. Optogenetic control shows that kinetic proofreading regulates the activity of the T cell receptor. *bioRxiv*. 432740 (2018). 10.1101/432740. 10.7554/eLife.42475 PMC6488296 30947807

[21] Xiong JP (2002). Crystal structure of the extracellular segment of integrin αVβ3 in complex with an Arg-Gly-Asp ligand. Science.

[22] Wang Y, Wang F, Wang R, Zhao P, Xia Q (2015). 2A self-cleaving peptide-based multi-gene expression system in the silkworm Bombyx mori. Sci. Rep..

[23] Barczyk M, Carracedo S, Gullberg D (2010). Integrins. Cell Tissue Res..

[24] Orlov AV (2014). Development of immunoassays using interferometric real-time registration of their kinetics. Acta Nat..

[25] Turner CE (2000). Paxillin and focal adhesion signalling. Nat. Cell Biol..

[26] Renshaw MW, Ren XD, Schwartz MA (1997). Growth factor activation of MAP kinase requires cell adhesion. EMBO J..

[27] Dupont S (2011). Role of YAP/TAZ in mechanotransduction. Nature.

[28] Elosegui-Artola A (2016). Mechanical regulation of a molecular clutch defines force transmission and transduction in response to matrix rigidity. Nat. Cell Biol..

[29] Chaudhuri O (2015). Substrate stress relaxation regulates cell spreading. Nat. Commun..

[30] Kim NG, Gumbiner BM (2015). Adhesion to fibronectin regulates Hippo signaling via the FAK-Src-PI3K pathway. J. Cell Biol..

[31] Müller K, Zurbriggen MD, Weber W (2014). Control of gene expression using a red- and far-red light-responsive bi-stable toggle switch. Nat. Protoc..

[32] Smith RW (2016). Unearthing the transition rates between photoreceptor conformers. BMC Syst. Biol..

[33] Müller K (2013). Multi-chromatic control of mammalian gene expression and signaling. Nucleic Acids Res..

[34] Reiser J (1996). Transduction of nondividing cells using pseudotyped defective high-titer HIV type 1 particles. Proc. Natl Acad. Sci. USA.

[35] Zhang XY, La Russa VF, Reiser J (2004). Transduction of bone-marrow-derived mesenchymal stem cells by using lentivirus vectors pseudotyped with modified RD114 envelope glycoproteins. J. Virol..

[36] Mitta B, Rimann M, Fussenegger M (2005). Detailed design and comparative analysis of protocols for optimized production of high-performance HIV-1-derived lentiviral particles. Metab. Eng..

[37] Schindelin J (2012). Fiji: an open-source platform for biological-image analysis. Nat. Methods.

[38] Bradski, G. The OpenCV Library. *Dr. Dobb’s J. Softw. Tools* (2000).

