# Supplementary material for: Optogenetic control of integrin-matrix interaction
Source: Commun Biol. 2019 Jan 8;2:15. doi: 10.1038/s42003-018-0264-7 (PMC6325061; doi:10.1038/s42003-018-0264-7)
Supplement: Supplementary file 1 — Description of Supplementary Video [file 42003_2018_264_MOESM1_ESM.docx]

**Description of Additional Supplementary Files**

**File Name**: Supplementary Movie 1

**Description**: HeLa cells stably expressing OptoIntegrin seeded on OptoMatrix were imaged while illumination with 660 nm light until adhesion was visible and subsequently illuminated with 740 nm. The time [min] and illumination condition are indicated in the video.

**File Name**: Supplementary Movie 2

**Description**: MCF7 cells stably expressing OptoIntegrin seeded on OptoMatrix were imaged while illumination with 660 nm light until adhesion was visible and subsequently illuminated with 740 nm. The time [min] and illumination condition are indicated in the video.

**File Name**: Supplementary Movie 3

**Description**: HEK-293T cells stably expressing OptoIntegrin seeded on OptoMatrix were imaged while illumination with 660 nm light until adhesion was visible and subsequently illuminated with 740 nm. The time [min] and illumination condition are indicated in the video.
