# Supplementary material for: Optogenetic control of integrin-matrix interaction
Source: Commun Biol. 2019 Jan 8;2:15. doi: 10.1038/s42003-018-0264-7 (PMC6325061; doi:10.1038/s42003-018-0264-7)
Supplement: Supplementary file 5 — Supplementary Information [file 42003_2018_264_MOESM5_ESM.pdf]

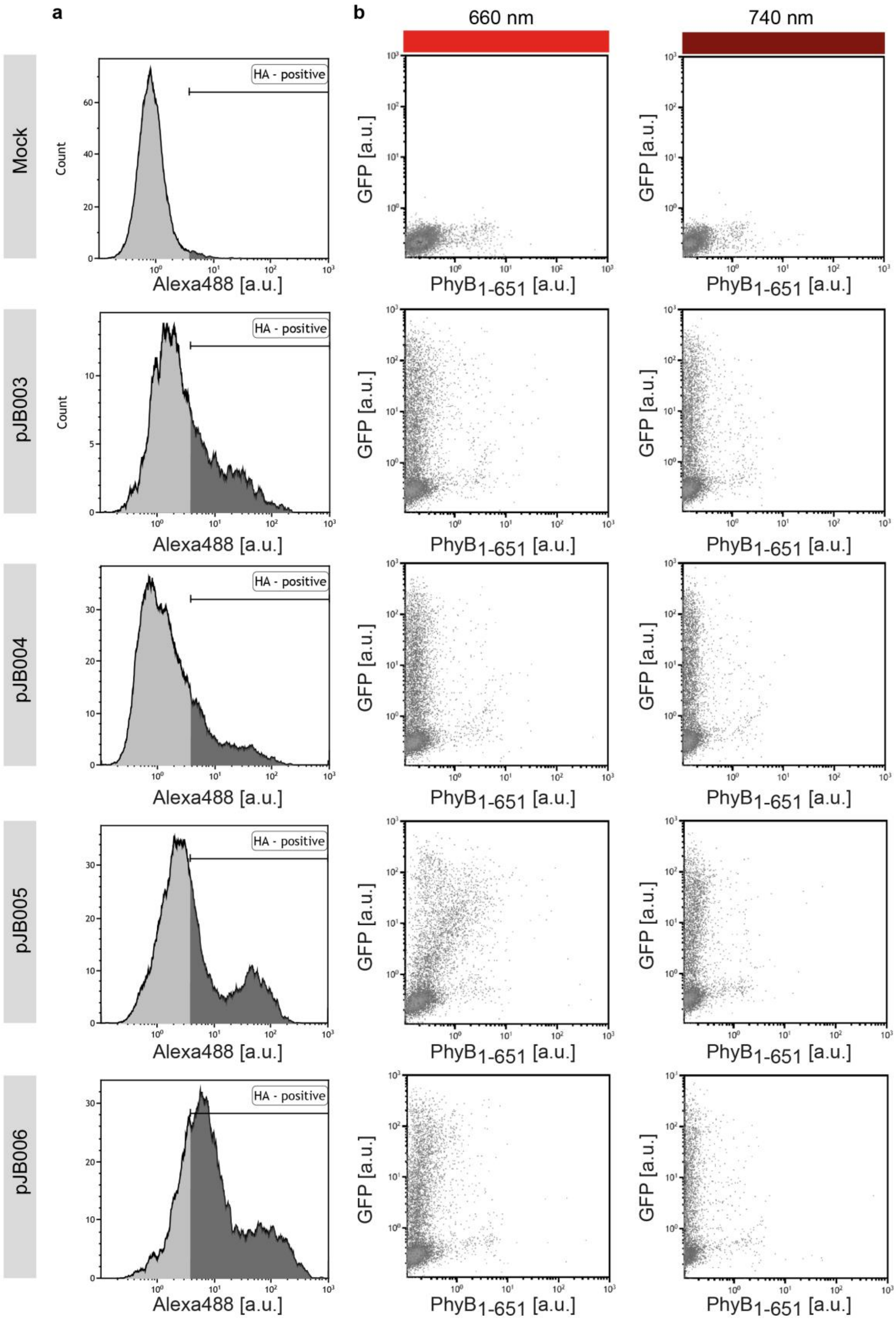

Supplementary Figure 1. Flow cytometric analysis of OptoIntegrin expression on the surface of HEK-293T cells transfected with the indicated plasmids. **(a)** Staining with a mouse anti-HA antibody and subsequently with an Alexa Fluor 488-labeled anti-mouse IgG (n > 5000 cells). **(b)** Cells co-transfected with GFP expression vector stained with $PhyB_{1\text{-}651}$ under 660 nm or 740 nm illumination.

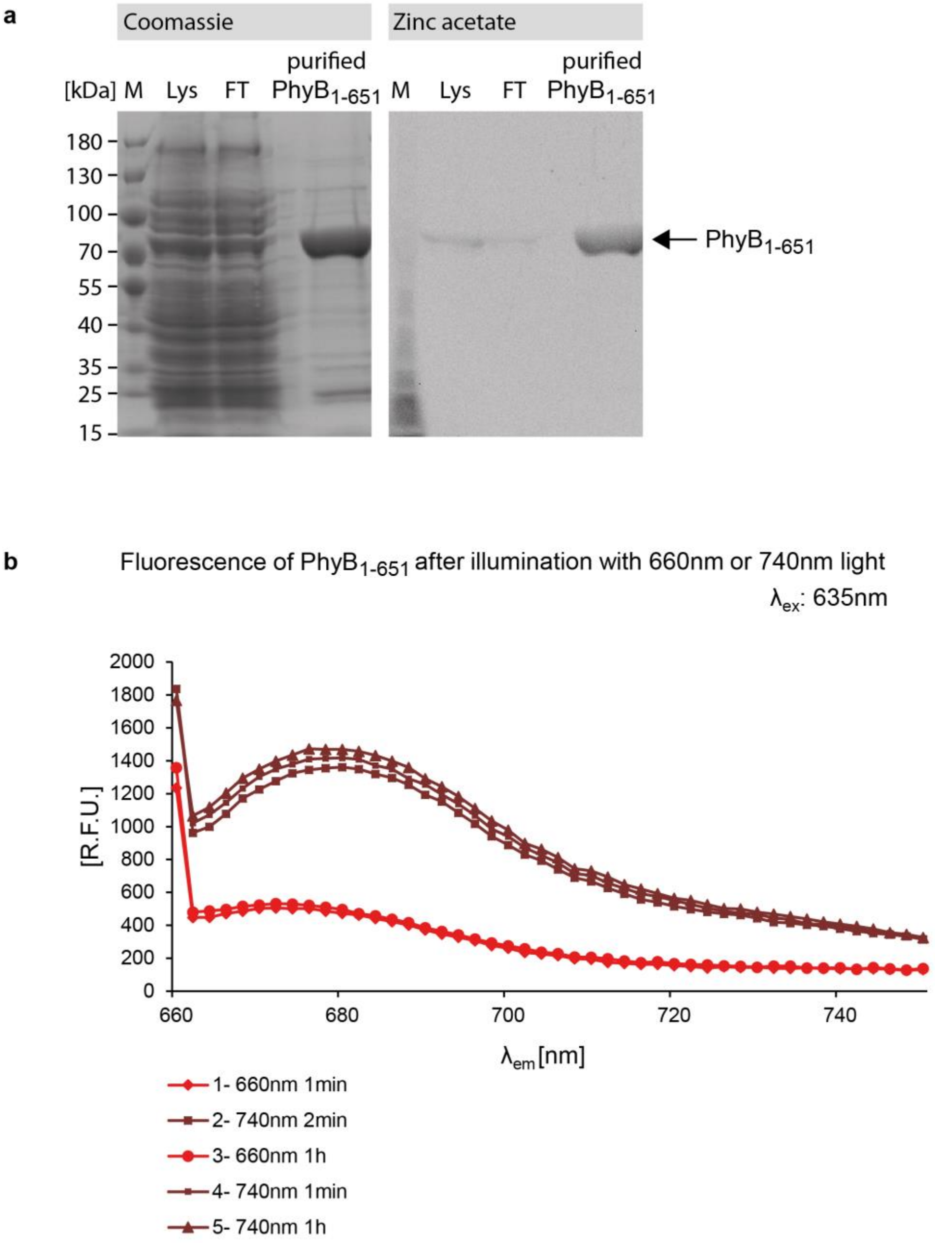

Supplementary Figure 2. PhyB production and characterization **(a)** Purification of PhyB$_{1-651}$. Left panel shows Coomassie staining for cleared cell lysate (Lys), flow through (FT) after IMAC and eluted PhyB$_{1-651}$. The right panel shows zinc acetate staining for the same gel to visualize binding of the chromophore phycocyanobilin to PhyB$_{1-651}$. **(b)** Fluorescence spectrum of PhyB. PhyB$_{1-651}$ (3 mg ml^{-1}) was illuminated for 1 minute with 660 nm (20 µmol m^{-2} s^{-1}) then for 2 minutes with 740 nm light (20 µmol m^{-2} s^{-1}) followed by 1 hour 660 nm, 1 minute 740 nm and 1 hour 740 nm illumination. The fluorescence emission spectra were subsequently measured after each step with excitation at λ_{ex}= 635 nm.

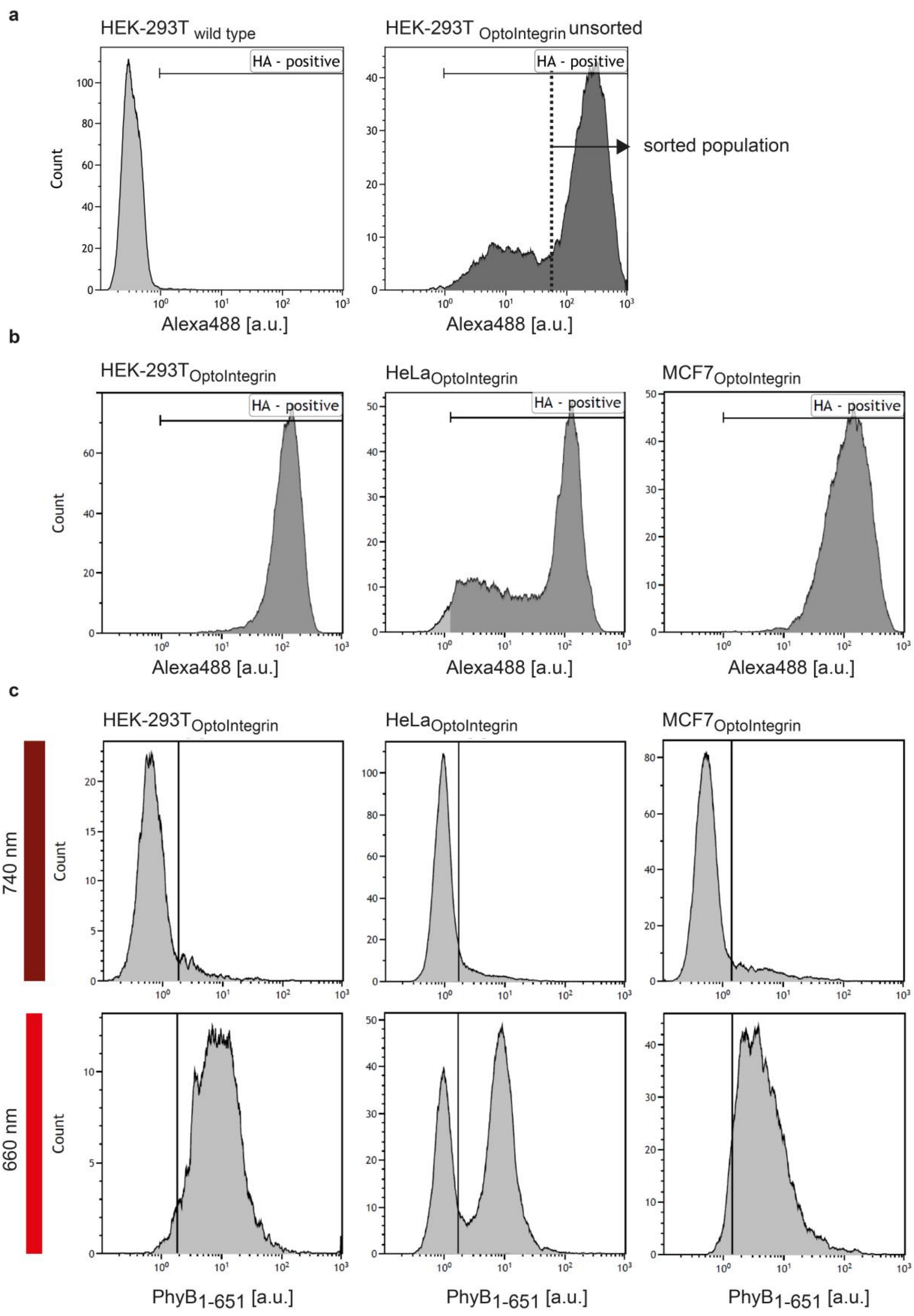

Supplementary Figure 3. Generation of cell lines stably expressing OptoIntegrin. **(a)** Sorting strategy for HEK-293T cells transduced with OptoIntegrin constructs as example for all cell lines. Cells transduced with OptoIntegrin-encoding lentiviral vectors were stained with a mouse anti-HA antibody and subsequently with an Alexa Fluor 488-labeled anti-mouse IgG. The left panel shows cells not

expressing the OptoIntegrin, the right panel shows the unsorted cells expressing OptoIntegrin. Only cells expressing high levels of OptoIntegrin (indicated with black arrow) were sorted for further use. **(b)** Staining of HA-tagged OptoIntegrin of all sorted cell lines. **(c)** $PhyB_{1-651}$ staining of sorted cell lines. Cells were incubated with $PhyB_{1-651}$ under 660 nm or 740 nm and subsequently analyzed for binding of $PhyB_{1-651}$ to the cells surface.

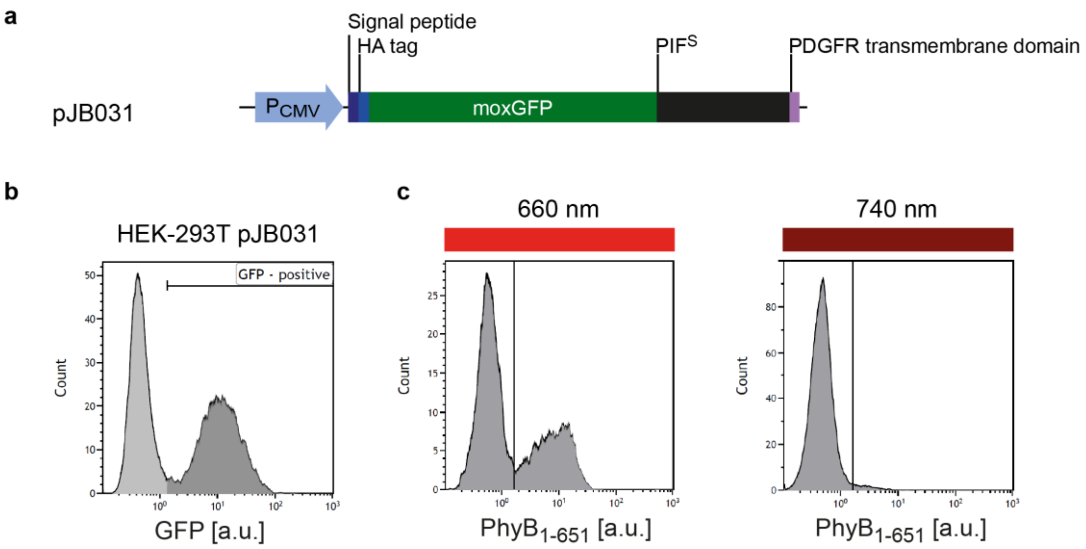

Supplementary Figure 4. Design and characterization of the control construct moxGFP-PIF^S. **(a)** Schematic representation of the expression construct. pJB031 contains a PIF^S linked to moxGFP and a platelet-derived growth factor receptor (PDGFR) transmembrane domain which allows display of the protein on the cell surface. **(b)** HEK-293T cells after transduction with construct pJB031 **(c)** PhyB$_{1\text{-}651}$ staining of HEK-293T cells transduced with pJB031. For this, cells were detached from the culture dish and incubated in a PhyB$_{1\text{-}651}$ solution (1.5 mg ml^{-1}) supplemented with 2% FCS for 1 hour at 4 °C under either 660 nm or 740 nm light (20 µmol m^{-2} s^{-1}). Before flow cytometric analysis of PhyB fluorescence, cells were washed and kept on ice.

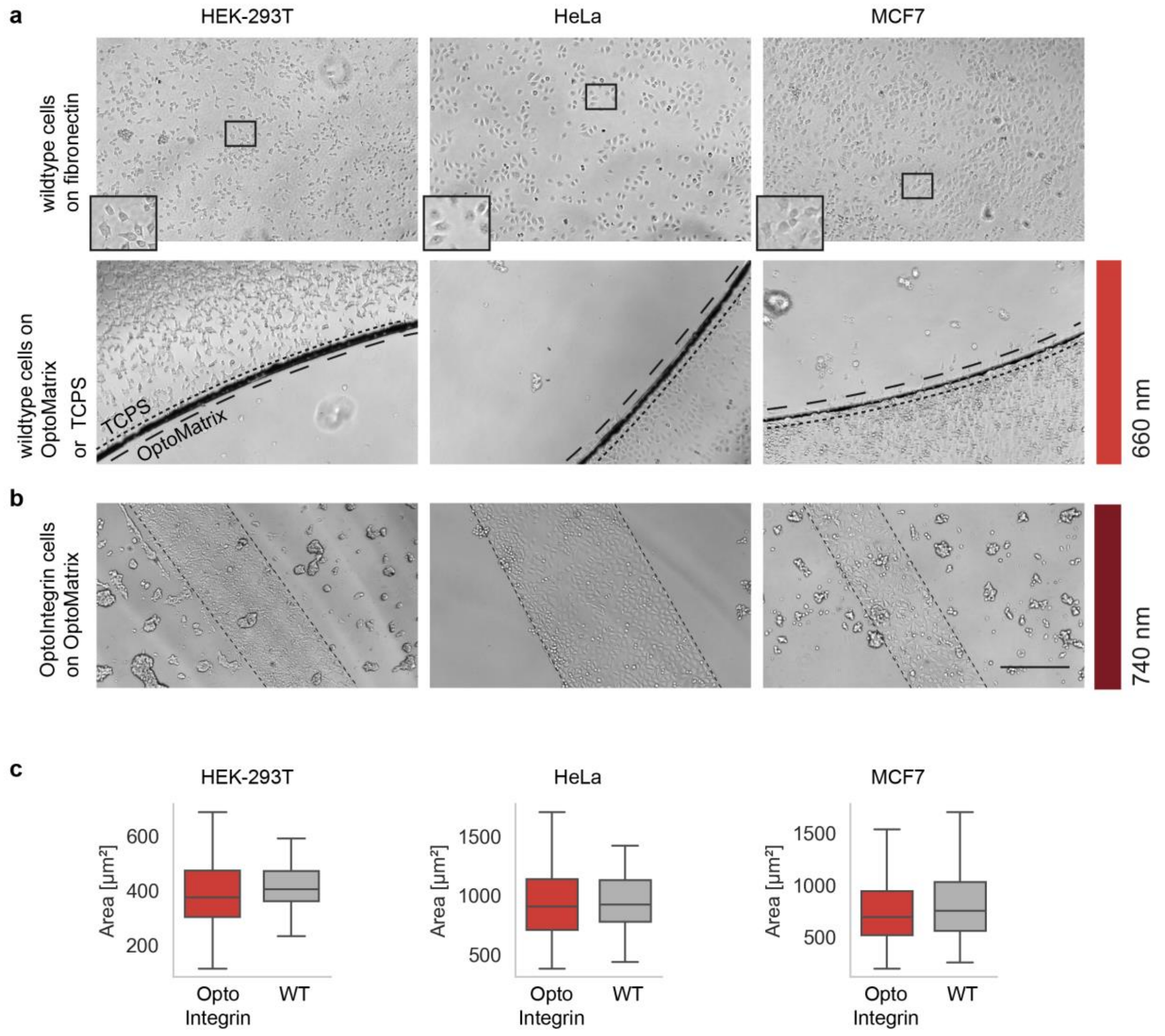

Supplementary Figure 5. Wildtype and OptoIntegrin-mediated Matrix attachment. **(a)** Attachment of wildtype cells. A fibronectin coated glass slide (top row) or a PhyB-coated glass slide (OptoMatrix)(bottom row) was placed into a tissue culture polystyrene (TCPS) well. Wildtype HEK-293T, HeLa and MCF7 cells were seeded and cell attachment on either substrate was evaluated after 5 h. **(b)** OptoMatrix-dependent attachment. $PhyB_{1\text{-}651}$ coating on the glass slides was disrupted by scratching with a 200 µl pipette tip prior to seeding OptoIntegrin-expressing cells. Scratched area indicated by dotted lines. (For (a) and (b) scale bar = 400 µm) **(c)** Analysis of cell spreading. OptoIntegrin expressing cells were cultivated for 5 h on OptoMatrix under 660 nm light (20 µmol m^{-2} s^{-1}) prior to quantifying cell-spreading area. As control, wildtype cells cultivated on fibronectin-coated slides were treated accordingly. Statistical analysis using Welch-test showed no significant differences between both conditions with $p = 0.184$ for HEK-293T, $p = 0.878$ for HeLa and $p = 0.124$ for MCF7.

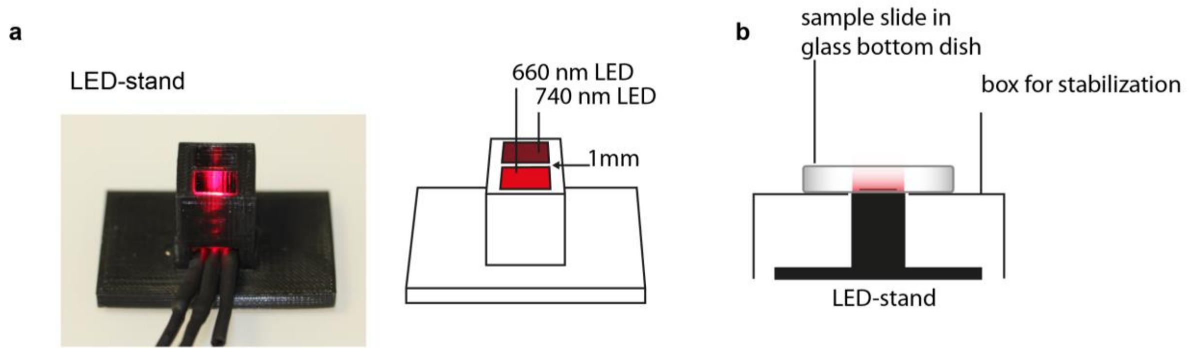

Supplementary Figure 6. Spatial control of attachment with LED-stand **(a)** Picture and schematic drawing of LED-stand **(b)** Side view of experimental set up

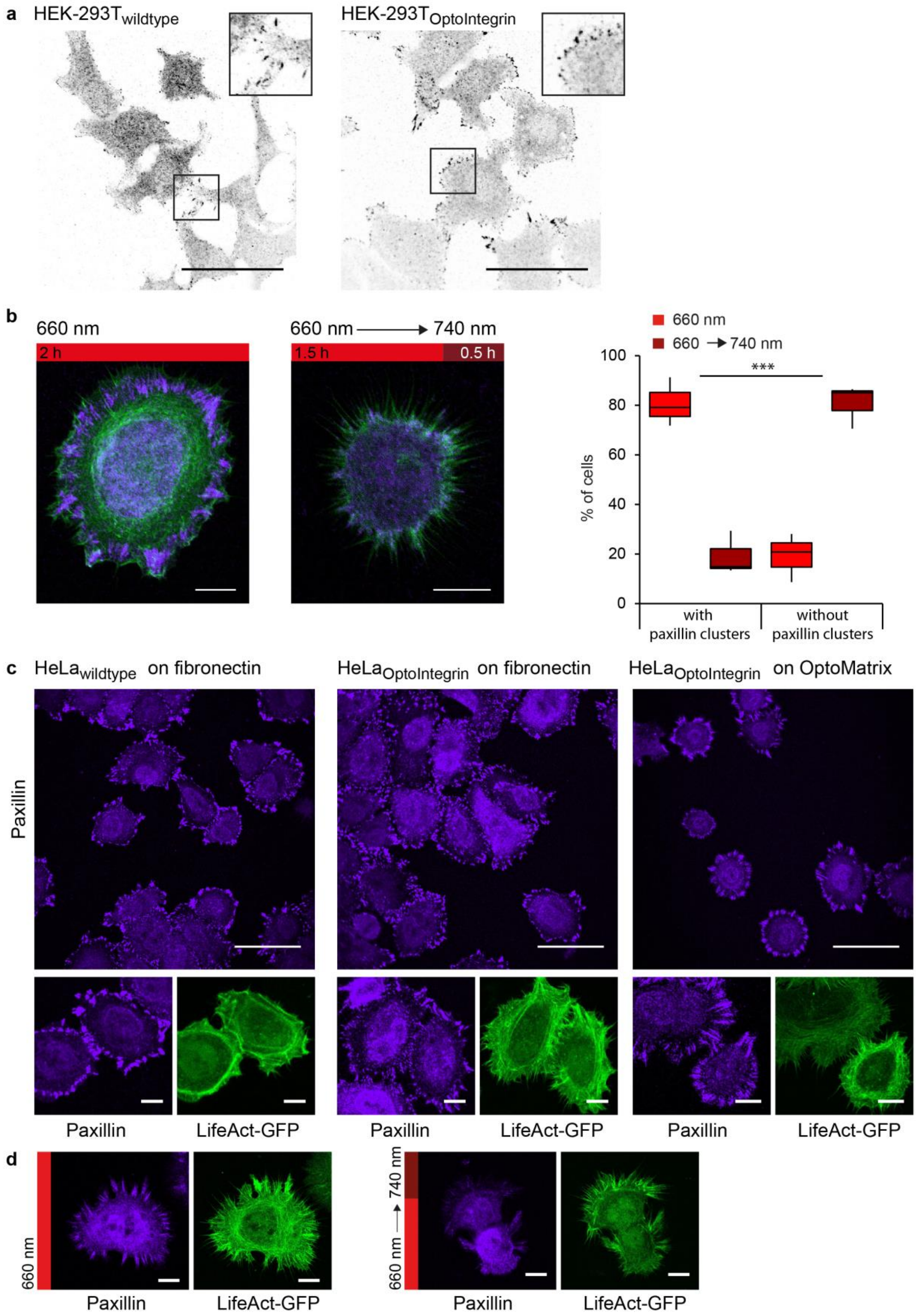

Supplementary Figure 7. Mechano-signalling in wildtype cells and OptoIntegrin-expressing cells. **(a)** Paxillin staining of wildtype and OptoIntegrin-expressing HEK-293T cells seeded on fibronectin-coated glass slides slips. Intensities of the pictures were inverted to better visualize clusters (scale bar = 50 µm). **(b/c/d)** Wildtype or OptoIntegrin-expressing HeLa cells were fixed and stained for Paxillin

(purple) with anti-paxillin antibody. The cells were additionally expressing LifeAct-GFP to visualize actin (green). **(b)** Cells were grown for 2 hours under 660 nm or 1.5 hour under 660 nm followed by 0.5 hours 740 nm before fixing and subsequent staining. The bar chart shows mean values ± standard deviation of three independent experiments where at least 30 cells per condition were analyzed ($p = 3{,}13*10^{-25}$ using Cochran-Mantel-Haenszel test with one degree of freedom, showing a consistent difference in the populations across several repeats) (scale bar = 10 µm). **(c)** Wildtype and OptoIntegrin-expressing HeLa cells were grown on OptoMatrix (under 660 nm illumination) or fibronectin-coated slides for 2-2.5 hours before fixation and staining (scale bar top row = 50 µm, scale bar bottom row = 10 µm). **(d)** Wildtype and OptoIntegrin-expressing HeLa cells were cultivated on OptoMatrix for 1.5 hours under constant 660 nm light. Subsequently, illumination was continued at 660 nm or switched to 740 nm for another 30 min (scale bar = 10 µm).

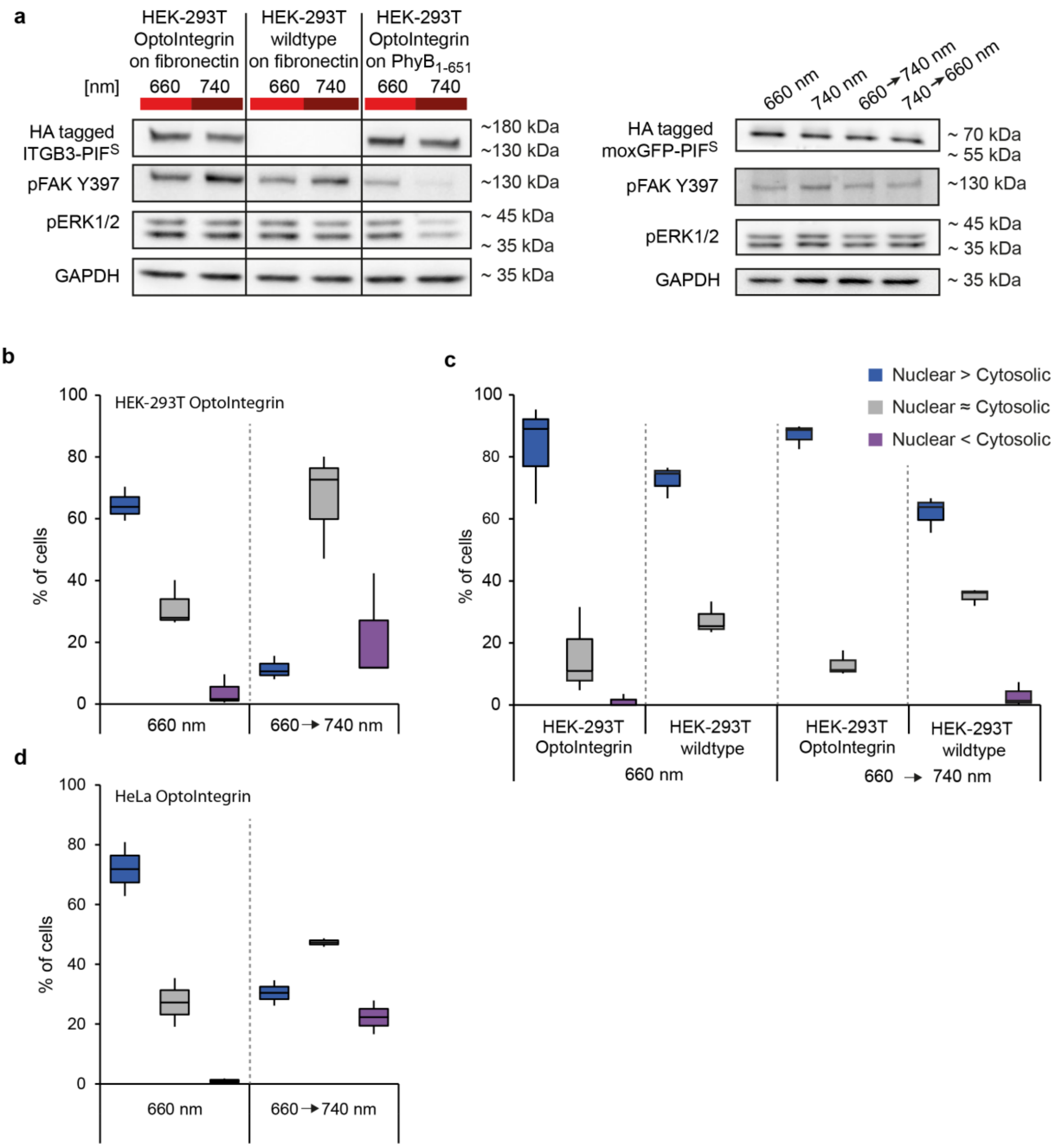

Supplementary Figure 8. Mechano-signalling in wildtype and OptoIntegrin expressing cells. **(a)** Light-dependent mechano-signalling. Left panel: HEK-293T wildtype or OptoIntegrin -expressing cells were seeded on fibronectin or OptoMatrix for 4 h under the indicated illumination conditions prior to analysis by Western blotting for OptoIntegrin (HA), focal adhesion kinase (FAK) phosphorylation and ERK1/2 phosphorylation. Right panel: HEK-293T moxGFP-PIF^S-expressing cells were seeded on OptoMatrix and illuminated for 4 h with constant 660 or 740 nm or switched after 3.5 hours to 740 or 660 nm, respectively, for 30 min prior to Western blot analysis. **(b)** YAP1 immunostaining of HEK-293T cells stably expressing OptoIntegrin seeded on OptoMatrix after 2 hours of constant illumination with 660 nm light, or after 1.5 hours of 660 nm light followed by 30 minutes of 740 nm. Data of three independent experiments is presented in box and whisker plot format, where n > 60 cells were counted per light condition. **(c)** YAP1 immunostaining of HEK-293T wildtype and OptoIntegrin expressing cells adhering to fibronectin-coated glass slide. Data of three experiments, where n > 40 cells were counted per condition is presented in box and whisker plot format. Statistical evaluation of each single

experiment using Chi-square test of independence showed p-values > 0.01 which we determined as non-significant. **(d)** YAP1 immunostaining of HeLa cells stably expressing OptoIntegrin. Data of two independent experiments, where n > 60 cells were counted per light condition, is presented in box and whisker plot format. I = 20 µmol m^{-2} s^{-1}

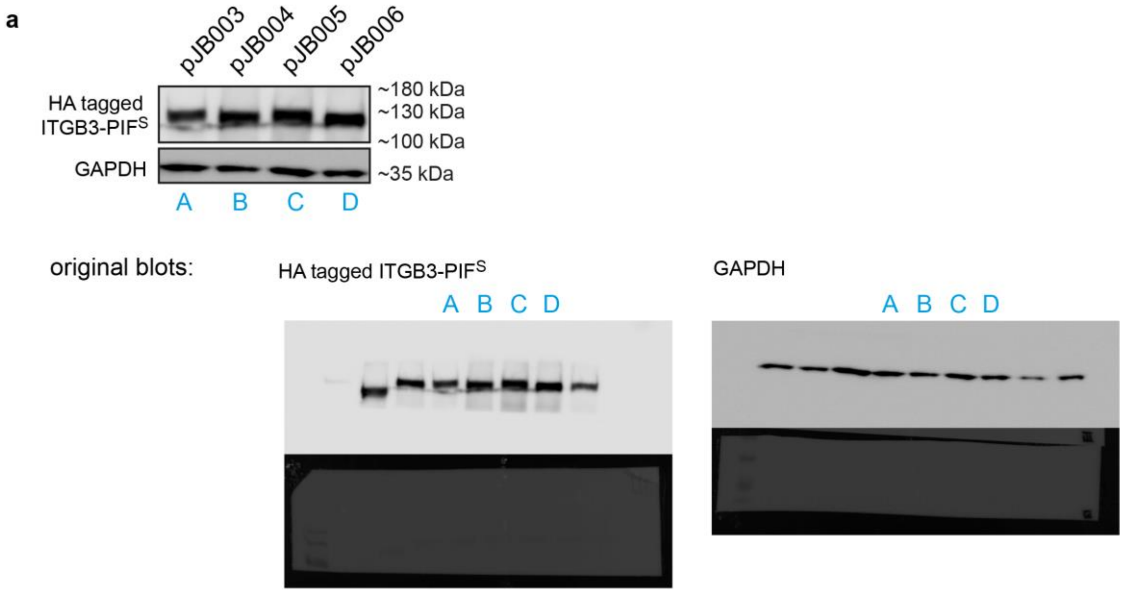

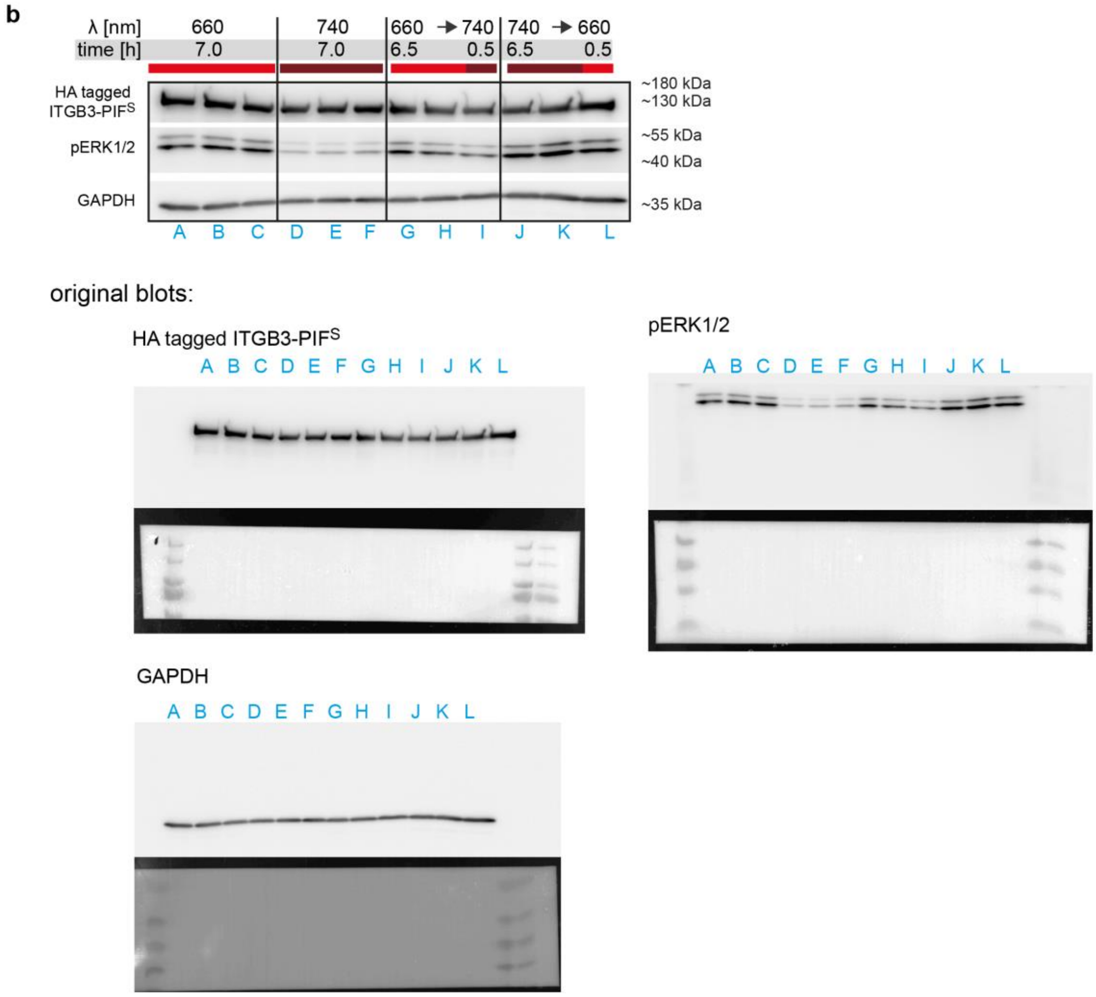

Supplementary Figure 9. Complete Western blots used in main text. **(a)** Blots corresponding to Figure 1d and **(b)** blots corresponding to Figure 3b.

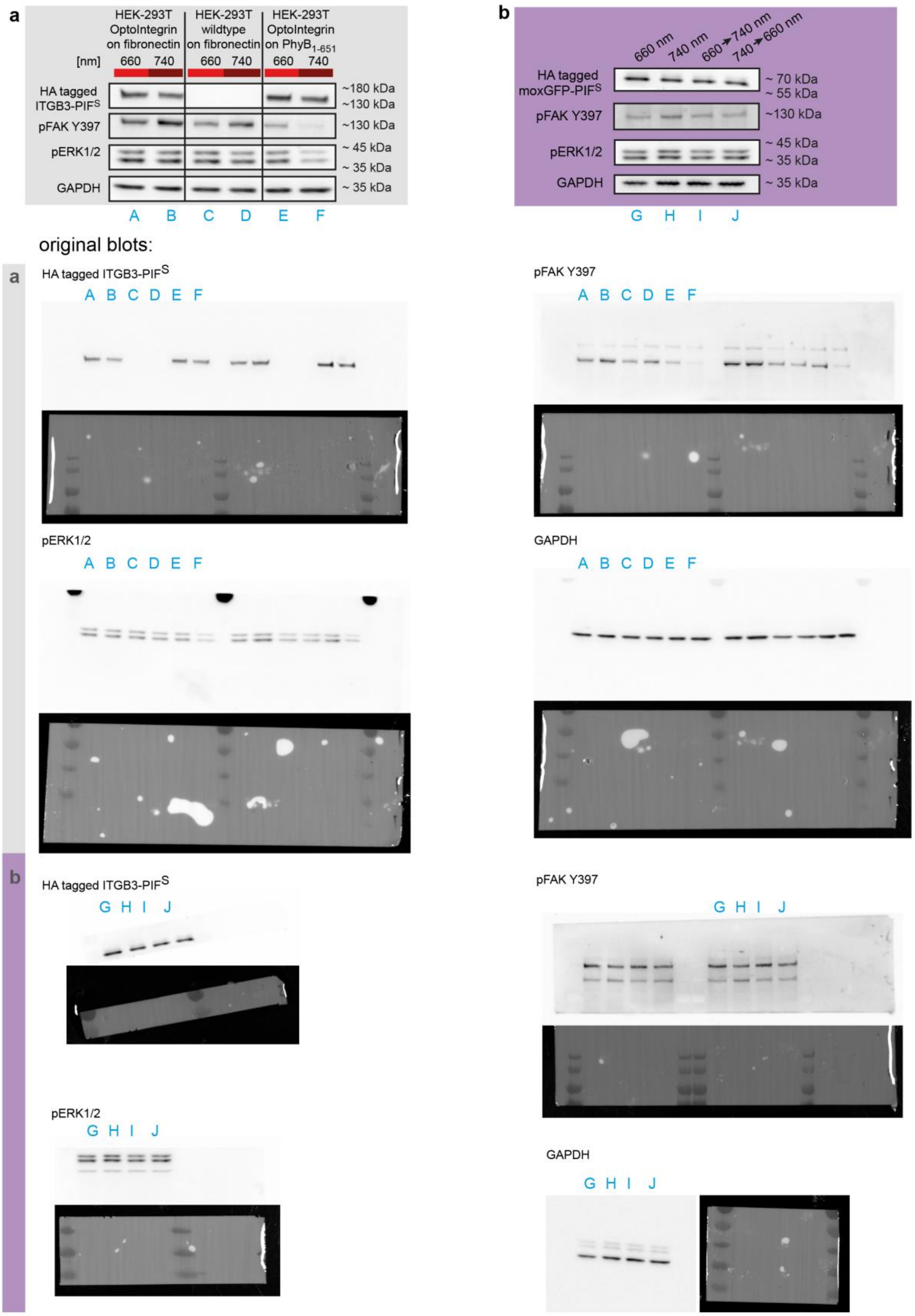

Supplementary Figure 10. Complete Western blots used in Supplementary Figure 8a. **(a)** Blots corresponding to left panel and **(b)** blots corresponding to right panel.

Supplementary Table 1. Plasmids designed in this work

pJB003	$P_{EF1\alpha}$-Signal Peptide-HA-1xSAG linker-(PIF^S (N-term)-3xSAG linker)-*ITGB3*-FLAG-P2A-*ITGAV*-pA<br><br>Coding sequences for *ITGB3* and *ITGAV* were obtained from pZeoSV-*ITGB3* and pcDNA3.1-*ITGAV*[1], respectively.<br><br>https://benchling.com/s/seq-4jg8JaOeIDoUcgkefZFa	**this work**
pJB004	$P_{EF1\alpha}$-SP-HA-1xSAG linker-(3xSAG linker-PIF^S (E108/G109)-3xSAG linker)-*ITGB3*-FLAG-P2A- SP -*ITGAV*-pA<br><br>https://benchling.com/s/seq-u69HNdUHznku2uqo6EVP	**this work**
pJB005	$P_{EF1\alpha}$-SP-HA-1xSAG linker-(3xSAG linker-PIF^S (M180/K181)-3xSAG linker)-*ITGB3*-FLAG-P2A- SP -*ITGAV*-pA<br><br>https://benchling.com/s/seq-OPIcRKR2ngZ8Vlt8kjNr	**this work**
pJB006	$P_{EF1\alpha}$-SP-HA-1xSAG linker-(3xSAG linker-PIF^S (S77/S78)-3xSAG linker)-*ITGB3*-FLAG-P2A- SP -*ITGAV*-pA<br><br>https://benchling.com/s/seq-7I85Z4ElQgHh3vpDN9K0	**this work**
pJB021	5'LTR - P_{CMV}-SP-HA-1xSAG linker-(3xSAG linker-PIF^S (M180/K181)-3xSAG linker)-*ITGB3*-FLAG-P2A- SP -*ITGAV*-3'LTR<br><br>https://benchling.com/s/seq-Xh42bsvR7etrTROMJtC1	**this work**
pJB031	5'LTR - P_{CMV}-SP-HA-1xSAG linker-moxGFP-1xGGSG-linker-PIF^S-3xSAG-linker-PDGFR transmembrane domain -3'LTR<br><br>https://benchling.com/s/seq-1cHvtKWwCwT3VcVTJy6W<br><br>moxGFP was first described in [2] and PDGFR-TM domain in [3]	**this work**

Supplementary Methods:

<u>Light boxes:</u>

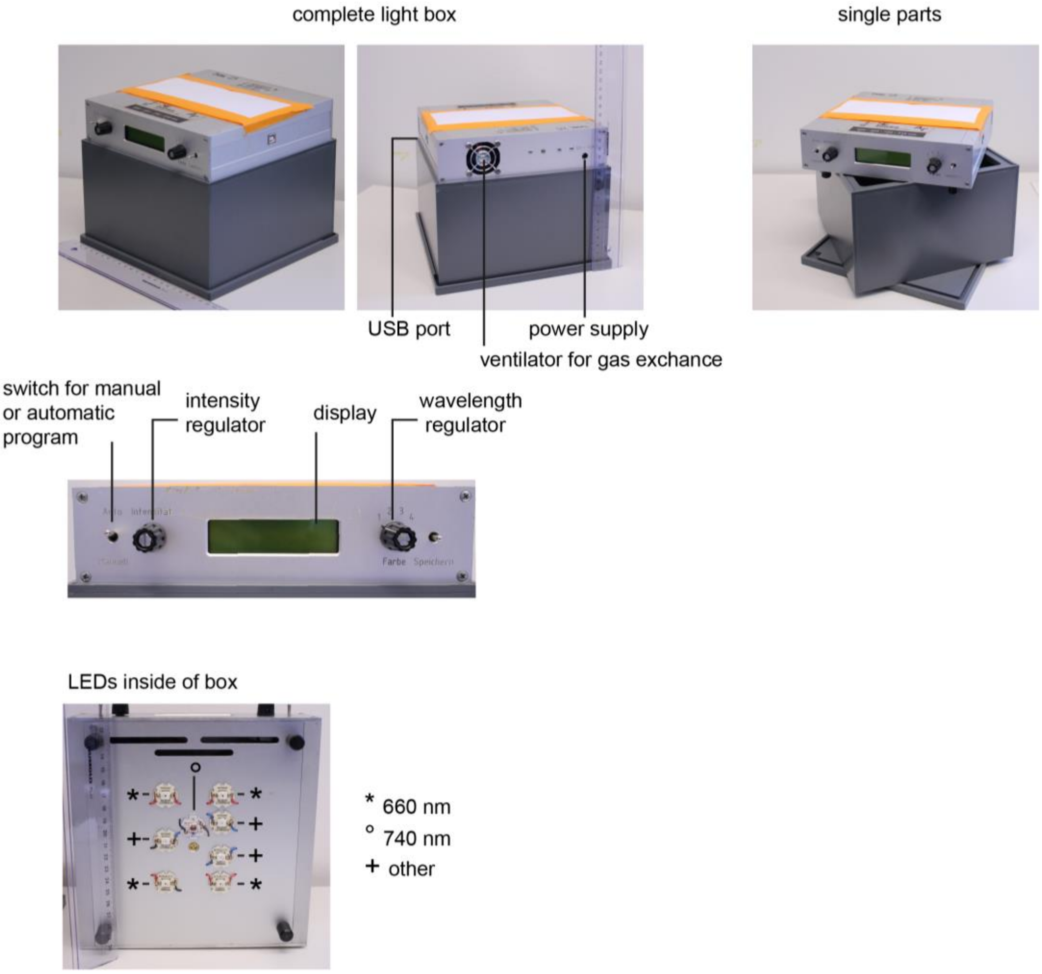

<u>Cell culture:</u>

Sources of cell lines:

- HEK-293T cells were purchased from DSMZ (Braunschweig).
- HeLa cells were a gift from B. Warscheid (Fischer et al., 2017, DOI: 10.1126/sciadv.1700475).
- MCF7 cells were a gift from R. Thünauer (Arbeithuber et al.,2015, DOI: 10.1155/2015/460598).

All cells were tested negative for mycoplasma using GATC MycoplasmaCheck (#SKU#B50400400).

<u>Flow cytometry and FACS:</u>

For cell sorting ProSort 1.5 software was used. For flow cytometry GalliosTM Flow Cytometer 1.2 software was used. For analysis of data Kaluza Analysis 1.5a was used.

For Flow cytometric experiments, gates were set in a way that control population showed less than 1% abundance inside set gates. For sorting, gates were set as indicated in the Supplementary Figure 3.

Coating of glass slides:

Chemical structure of GLYMO, after coating reaction:

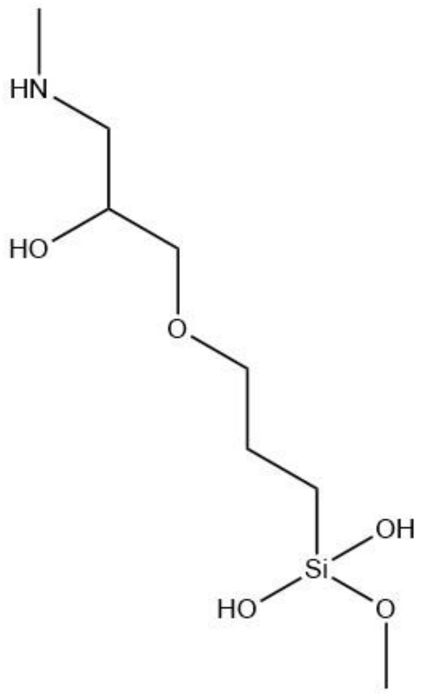

Statistical analysis:

Python code used for Welch-test:

```
from scipy.stats import ttest_ind
print(ttest_ind(PHYB["Area [µm²]"], Fibro["Area [µm²]"], equal_var=False), "Welch-test")
```

where PHYB["Area [µm²]"] and Fibro["Area [µm²]" indicate two arrays with the determined values of the corresponding cell areas

Supplementary References

1. Ahrens, I. G. *et al.* Evidence for a differential functional regulation of the two β3-integrins αVβ3 and αIIbβ3. *Exp. Cell Res.* **312,** 925–937 (2006).

2. Costantini, L. M. *et al.* A palette of fluorescent proteins optimized for diverse cellular environments. *Nat. Commun.* **6,** 7670 (2015).

3. Gronwald, R. G. K. *et al.* Cloning and expression of a cDNA coding for the human platelet-derived growth factor receptor: Evidence for more than one receptor class. *Cell Biol.* **85,** 3435–3439 (1988).
